# Chemical Glycosylation and Its Application to Glucose Homeostasis-Regulating Peptides

**DOI:** 10.3389/fchem.2021.650025

**Published:** 2021-04-12

**Authors:** Chaitra Chandrashekar, Mohammed Akhter Hossain, John D. Wade

**Affiliations:** ^1^Florey Institute of Neuroscience and Mental Health, University of Melbourne, Melbourne, VIC, Australia; ^2^School of Chemistry, University of Melbourne, Melbourne, VIC, Australia

**Keywords:** glucose homeostasis-regulating peptides, glycosylation, Insulin, pharmacokinetics, solid phase peptide synthesis

## Abstract

Peptides and proteins are attractive targets for therapeutic drug development due to their exquisite target specificity and low toxicity profiles. However, their complex structures give rise to several challenges including solubility, stability, aggregation, low bioavailability, and poor pharmacokinetics. Numerous chemical strategies to address these have been developed including the introduction of several natural and non-natural modifications such as glycosylation, lipidation, cyclization and PEGylation. Glycosylation is considered to be one of the most useful modifications as it is known to contribute to increasing the stability, to improve solubility, and increase the circulating half-lifves of these biomolecules. However, cellular glycosylation is a highly complex process that generally results in heterogenous glycan structures which confounds quality control and chemical and biological assays. For this reason, much effort has been expended on the development of chemical methods, including by solid phase peptide synthesis or chemoenzymatic processes, to enable the acquisition of homogenous glycopeptides to greatly expand possibilities in drug development. In this mini-review, we highlight the importance of such chemical glycosylation methods for improving the biophysical properties of naturally non-glycosylated peptides as applied to the therapeutically essential insulin and related peptides that are used in the treatment of diabetes.

## Introduction

**Figure 1:**
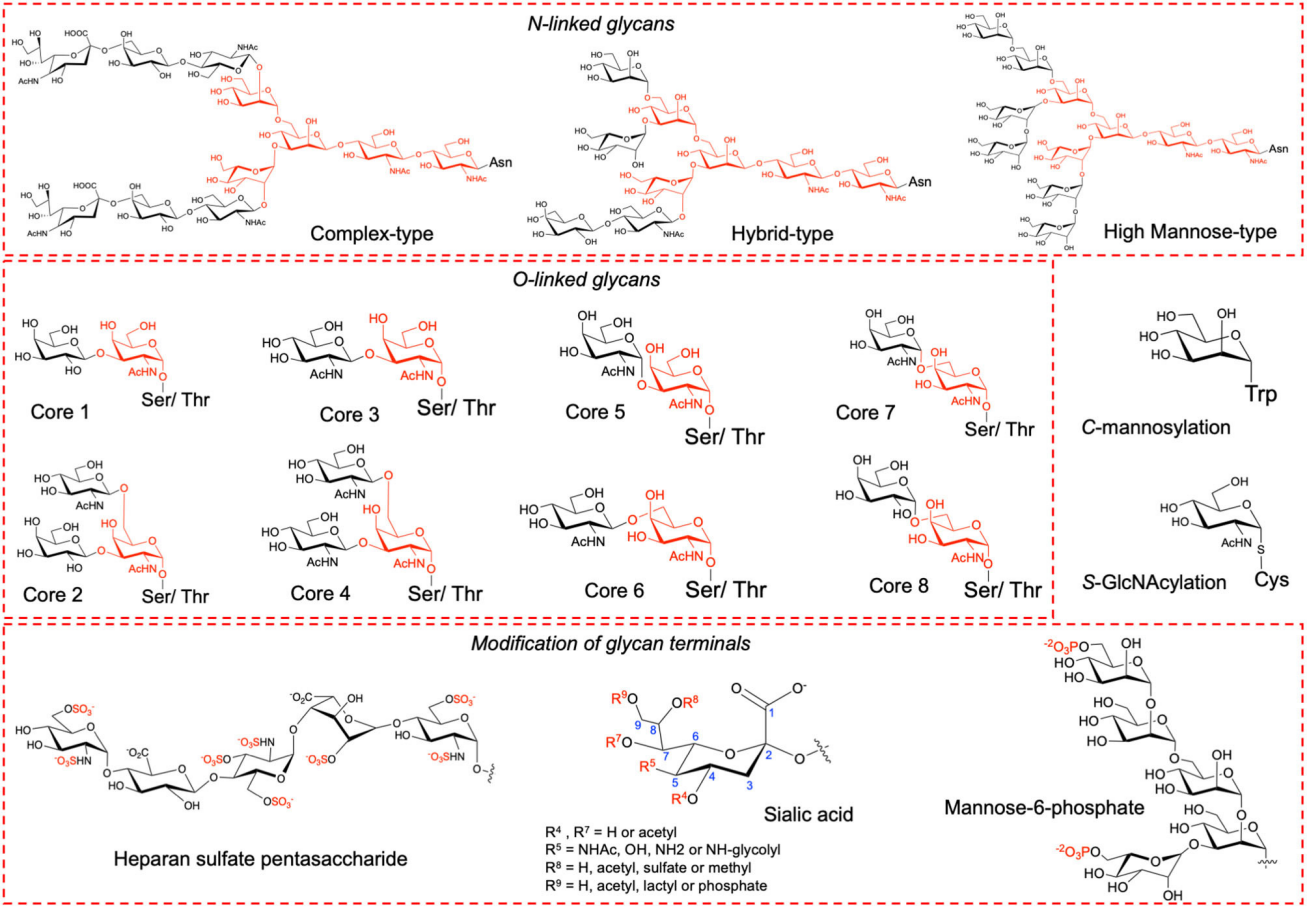
Types of glycosylation and of glycans modified at terminals.

Proteins undergo several types of post-translational modifications (PTMs) of which glycosylation is the most abundant type among eukaryotes. Glycosylation confers considerable physiological and biological effects including stability, folding, solubility, trafficking, immunogenicity, cell growth, cell-cell adhesion and cell-pathogen interactions (Varki, 2017). Glycosylation can be classified into *N*-, *O*-, *C*- or *S*-linked based upon the amino acid side chain atoms to which the oligosaccharide is attached (Maynard et al., 2016). The most prevalent types are *N*- and *O*-linked glycosylation where the side chain of Asn in the consensus sequence Asn-Xaa-Ser/Thr (Xaa is any amino acid except Pro) and the side chain of either Ser or Thr is modified, respectively (Figure 1). Less common are *C*-linked and *S*-linked glycosylation where the Trp and Cys side chains are modified, respectively (Figure 1). In humans, *N*-linked glycosylation is of three major types, namely, high mannose, hybrid and complex type each with a common pentasaccharide core (Man_3_GlcNAc_2_). *O*-linked glycosylation comprises of eight types, namely, core 1 to core 8, all having a common GalNAc-α-1-*O*-Ser/Thr linkage. The glycan terminals can undergo further modifications such as acetylation, methylation, sulfation, phosphorylation, and sialylation (Figure 1) that alters protein's surface charge and isoelectric point (pI) (Solá and Griebenow, 2010; Muthana et al., 2012).

Glycopeptides and proteins can be obtained by either recombinant expression or chemical methods. Various eukaryotic and prokaryotic expression systems such as mammalian cell lines, insects, yeasts and bacteria can be engineered to obtain the desired glycosylation pattern (Schmidt, 2004). Plants are also emerging as powerful expression systems (Burnett and Burnett, 2020). However, the processing of *N*-glycans in the above expression systems generally results in other non-human PTMs that are immunogenic to humans. To address this, pharmaceutical companies are now developing human cell lines in order to produce human-like modifications (Chin et al., 2019). Glycoprotein therapeutics produced by human embryonic kidney HEK293 and fibrosarcoma HT-1080 cell lines are now been approved by US Food and Drug Administration (FDA) and European Medicines Agency (EMA) (Dumont et al., 2016). Chemical approaches provide access to more defined and precise glycan structures which includes chemoenzymatic remodeling, total chemical synthesis and semisynthesis (Thompson and Muir, 2020). Chemoenzymatic remodeling primarily employs endo-β-*N*-acetyl-glucosaminidases (ENGases) and their mutants that lack inherent hydrolase activity. The mutant ENGase brings about transglycosylation between a pre-assembled glycan donor and a peptide or protein substrate with a single *N*-acetylglucosamine (GlcNAc). Semisynthesis involves both, the chemical synthesis of smaller peptide fragment with desired modification and recombinant expression of remaining larger fragment(s), thus exploiting the advantages of both the methods. For smaller proteins, total chemical synthesis is the most flexible and robust approach for introducing any number and kind of modification at virtually any position. A glycosylated amino acid building block can be directly incorporated during solid phase peptide synthesis (SPPS) or, alternatively, a short glycan is first introduced which is later extended by chemical or enzymatic methods (Unverzagt and Kajihara, 2018). In this mini review, we highlight the chemical non-native glycosylation methods as specifically applied to glucose homeostasis-regulating peptides involved in the treatment of diabetes including insulin, glucagon, glucagon-like peptide-1, and pramlintide.

## Glycosylation of Therapeutically Valuable Diabetes Peptide Drugs

The primary issue for peptide and protein-based therapeutics is their susceptibility to proteases which limits their administration only by parenteral route which is inconvenient when patients may require multiple injections per day, for example, insulin for type 1 diabetes. Industrially, there are other challenges that arise due to their physical and chemical instabilities during several stages of production, purification, storage and delivery (Manning et al., 2010; Pham and Meng, 2020). This can result in denaturation, aggregation, precipitation, surface adsorption, chemical degradation which changes their pharmacological properties which can result in inactive or poorly active products. Various strategies are routinely used to stabilize the protein either by introducing chemical modifications to protein structure or by using external stabilizing agents (Otvos and Wade, 2014). Some of the chemical modifications include PEGylation, lipidation, glycosylation, cyclization, and non-natural amino acids. Glycosylation, being a natural modification, is known to improve the pharmacodynamics and pharmacokinetics profile without leading to immunogenicity (Walsh and Jefferis, 2006; Solá and Griebenow, 2010). The versatility of glycosylation has also led to the use of carbohydrates as temporary hydrophilic side chain protecting group for recombinant peptides (Chandrashekar et al., 2019). This results in an increase in the solubility and physical stability of hydrophobic and aggregation prone peptide and allows selective introduction of glycans and other non-natural modifications.

**Figure 2:**
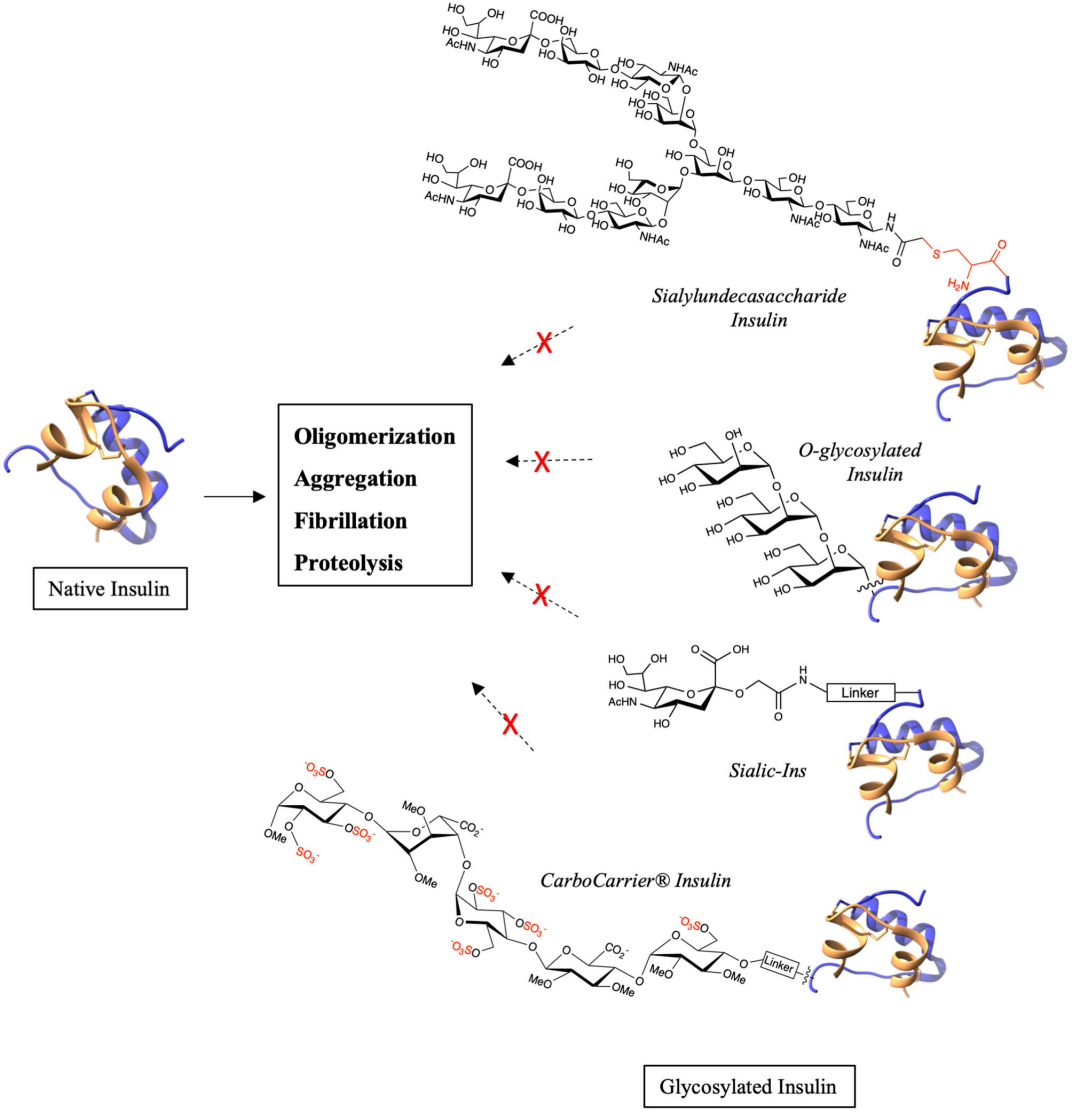
Examples of chemical glycosylation that stabilizes native insulin.

Diabetes affects millions of people worldwide and is classified into type 1, type 2 and gestational diabetes. Depending upon the medical condition of the patient, one or more of insulin, glucagon, GLP-1, exenatide, pramlintide are used for treating the condition. However, these peptides and proteins are highly susceptible to oligomerization and fibrillation which lead to major storage and handling issues and also immunogenicity (Akbarian et al., 2020). Much effort has been made to address these challenges and to reduce the associated side effects. This has been best illustrated by studies on insulin (Akbarian et al., 2020; Østergaard et al., 2020). This hormone has dramatically aided the treatment of diabetic patients, especially for people with type 1 who solely depend on insulin and in type 2 when other treatment methods fail. Various glycosylated insulin analogs have been investigated by scientists in order to improve their pharmacodynamics and pharmacokinetics (Baudyš et al., 1995; Uchio et al., 1999). Recently, a naturally *O*-glycosylated human insulin at ThrB27 has been discovered by targeted mass spectrometry (Yu et al., 2017). A separate investigation of chemically synthesized *O*-glycosylated insulin analogs (Guan et al., 2018) showed these to possess increased *in vitro* stability. Using total chemical synthesis, 12 glycosylated variants with GalNAc, mono-, di- and tri-mannose were synthesized by varying the attachment position (Guan et al., 2018). The analogs having *O*-mannosylation were found to have enhanced proteolytic stability and decreased oligomerization propensity especially, the analog with tri-mannosylation at ThrB27 (Figure 2). Thus, the presence of *O*-glycan at position ThrB27 in both naturally occurring, post-translationally modified insulin and obtained through artificial chemical synthesis confirmed the positive influence of glycan on the hormone's conformation and stability.

Modification of therapeutic antibodies with terminal sialic acid is known to improve the serum half-life and stability due to the negative charge which reduces its affinity for receptors in liver (Higel et al., 2019; Saunders, 2019). Similar results have been observed when glucose homeostasis-regulating peptides are modified with sialic acid. A long-acting insulin analog was obtained by modification of insulin with dendritic sialyl oligosaccharide by an enzymatic method (Sato et al., 2004). In addition, insulin modified with sialylundecasaccharide (Figure 2) demonstrated excellent stability against fibrillation at high concentration and high temperature (Hossain et al., 2020). Using total chemical synthesis, insulin possessing an additional, orthogonally-protected Cys at the *N*-terminal of B-chain was synthesized to which a sialyloligosaccharide was selectively introduced. This sialo-glycoinsulin is an example of *S*-glycosylation, which, importantly, maintained native structure and *in vivo* blood glucose lowering activity (Hossain et al., 2020). Moreover, an *S*-glycan is reported to be more stable toward proteolysis than analogs of *O*-glycan (De Leon et al., 2017). The importance of sialyl modification was further demonstrated through the synthesis of Sialic-Ins (Kabotso et al., 2020). Sialic acid conjugated to a small peptide was introduced via a disulfide bond to the thiopropyl linker that is attached to PheB1 (Figure 2). Each of the analogs containing 1–4 sialic acid moieties had similar *in vivo* activities to native human insulin. Their stability toward fibrillation also increased from 2 days for native insulin to up to 4 weeks for Sialic-Ins. Remarkably, just a single sialic acid was enough to stabilize insulin while maintaining its structure and bioactivity. Thus, sialo-glycoinsulin can be of potential benefit for use in insulin pumps where fibrillation leads to dosage problems and associated side effects. In addition to insulin, sialyl modification of glucagon and glucagon-like peptide 1 (GLP-1) has also shown promising results. Glucagon is a 29 amino acid containing peptide that increases the blood glucose levels and is used in treatment of hypoglycemia in diabetic patients. It is highly unstable and forms fibrils rapidly in aqueous solution (Stigsnaes et al., 2007). A glucagon containing a sialyl-complex type oligosaccharide at Asn28 obtained by chemoenzymatic method (Higashiyama et al., 2018) showed higher protease resistance and bioactivity compared to native glucagon. GLP-1 stimulates insulin secretion in a glucose-dependent manner and is now an increasingly-used therapeutic for treating type 2 diabetes. However, GLP-1 is rapidly cleared in the body principally by two proteolytic enzymes, DPP-iV and NEP 24.1 (Plamboeck et al., 2005), and is also prone to aggregation due to physical instability. The influence of glycosylation with respect to its number, position, charge and size on GLP-1 was investigated (Ueda et al., 2009). Homogeneous analogs with GlcNAc, LacNAc and sialyl LacNAc obtained by the chemoenzymatic method demonstrated improved protease stability and bioactivity, with sialyl LacNAc modification to be the most effective. Increasing the number of sialic acids increased the protease stability, thus implying extended plasma half-life and bioactivity. Similarly, glycosylation of exendin-4, a GLP-1 receptor agonist, with sialyl LacNAc at Asn28 by glycosyltransferase increased its proteolytic stability and prolonged the *in vivo* blood-glucose lowering activity (Ueda et al., 2010).

Interestingly, insulin and GLP-1 were each modified by a novel CarboCarrier® technology bearing a sulfated pentasaccharide (De Kort et al., 2008; Irwin et al., 2015). The pentasaccharide specifically binds to plasma protein antithrombin III (ATIII) thereby increasing the plasma half-life of the conjugated protein at sub-anticoagulant concentrations. Insulin analogs with sulfated pentasaccharide at either PheB1 or LysB29 were obtained by modification of recombinant-derived human insulin by thiol-maleimide chemistry using ethylene glycol spacer (Figure 2). The analogs had high solubility and stability against aggregation. Pharmacokinetic studies in rat, mouse and dog models showed that the half-life of the insulin analogs increased greatly. They further studied the pharmacokinetic and pharmacodynamic properties of CarboCarrier® insulin analog modified at LysB29 (SCH 900948) in humans (Miltenburg et al., 2013) and their phase 1 studies confirmed an extended half-life when compared to endogenous insulin. The CarboCarrier® technology was also applied to a D-Ala^8^GLP-1 analog to produce 17 novel long-acting GLP-1 conjugates (Irwin et al., 2015). GLP-1 was modified at three positions by varying the length and chemical nature of the linker. While all the conjugates had potent insulin releasing activity, the conjugate at Lys37 with a short linker exhibited similar or better bioactivity than other GLP-1 agonist such as exenatide or liraglutide.

Amylin is another glucose homeostasis-regulating peptide hormone that contains 37 amino acids and contributes to the control of post-prandial glucose levels. Pramlintide (Symlin), a stable analog of amylin, is clinically used as an adjunctive treatment along with insulin (Grunberger, 2013). The naturally occurring *O*-glycosylated amylin is reported to be inactive, however, modification of pramlintide by *N*-glycosylation (Tomabechi et al., 2013; Kowalczyk et al., 2014) maintained the bioactivity. A library of *N*-glycosylated pramlintide analogs with GlcNAc, Man_3_(GlcNac)_2_ and complex type sialyl oligosaccharide at each of the available six Asn residues were investigated. The amylin-responsive receptor activity using pramlintide as control revealed that size of the glycans and the activity were inversely related and glycans were tolerated well toward the *C*-terminal. The Brimble group further studied analogs with GlcNAc moieties at Asn21 and/or Asn35 attached either via native linkage or triazole linkage through click chemistry (Yule et al., 2016). Activity assays on the amylin and pramlintide receptor, AMY_1(a)_, demonstrated that GlcNAc at Asn21 and Asn35 either through *N*-glycosylation, click chemistry or both, maintained their activity. Thus, the introduction of non-native glycosylation at an optimum site of a protein without effecting conformation and receptor binding properties has been consistently shown to significantly improve its pharmacokinetic and pharmacodynamic properties.

## Concluding Remarks

Glycosylation is an ubiquitous post-translational modification that plays a major role in the structural and functional properties of peptides and protein. Glycosylation of therapeutic peptides and proteins endows a significant positive impact on their pharmacological properties such as solubility, stability, bioavailability, receptor specificity and immunogenicity. Its introduction by chemical methods has been developed to a point where it is now readily feasible to design and synthesize a variety of desired analogs. The critical factors while introducing non-native glycosylation are the site of introduction, size, number and the charge of glycan. These factors not only favor the pharmacological properties but may also adversely modify the structural features of proteins, sometimes making it unavailable for receptor binding. Glucose homeostasis-regulating peptide hormones are easily prone to oligomerize and undergo fibrillation, long-standing side reactions that can be ameliorated by glycosylation. The significant potential of glycosylation has thus been highlighted through the successful modification of these clinically important peptides. These positive findings augur well for glycosylation to be more routinely employed as a general modification to improve the physicochemical and biological features of therapeutic peptides.

## Author Contributions

All authors equally made significant intellectual and written contribution to the work and have approved it for publication.

## Conflict of Interest

The authors declare that the research was conducted in the absence of any commercial or financial relationships that could be construed as a potential conflict of interest.
